# Mucoadhesive polydopamine-coated nanoparticle-mediated inner ear drug delivery for hearing loss treatment

**DOI:** 10.1186/s12967-025-07103-z

**Published:** 2025-10-08

**Authors:** Subin Kim, Seo Young Cheon, Keum-Jin Yang, Seong Su Won, Simin Chun, Hyorim Nam, Dong-Kee Kim, Heebeom Koo

**Affiliations:** 1 Department of Otolaryngology-Head and Neck Surgery, College of Medicine, Soonchunhyang University, Cheonan, Republic of Korea; 2 Department of Otolaryngology–Head and Neck Surgery, Daejeon St. Mary’s Hospital, College of Medicine, The Catholic University of Korea, Daeheung-dong, Jung-gu, Daejeon, Republic of Korea; 3 Department of Medical Life Sciences and Department of Medical Sciences (Graduate School), College of Medicine, The Catholic University of Korea, 222 Banpo-daero, Seocho-gu, Seoul, 06591 Republic of Korea

**Keywords:** Mucoadhesive, Nanoparticle, Polydopamine, Intratympanic injection, Inner ear drug delivery

## Abstract

This study investigated the potential of mucoadhesive polymeric nanoparticles coated with polydopamine (Dopa-NPs) for inner ear drug delivery. Dopa, inspired by mussel adhesion proteins, leveraged the mucoadhesive properties of NPs and enhanced their retention within the cochlea. Dopa-NPs were compared with non-adhesive uncoated control NPs to reveal their safety and drug delivery efficiency. The safety evaluation demonstrated no toxicity during in vitro test using HEI-OC1 cells and in vivo test using mouse with auditory function assessment. When coumarin-encapsulated NPs were administered through intratympanic injection to mice, Dopa-NPs provided intense fluorescence in the inner ear compared to non-adhesive control NPs. Furthermore, dexamethasone (Dex)-encapsulated Dopa-NPs exhibited significantly higher drug concentrations in the cochlea than dexamethasone sodium phosphate and uncoated NPs. Finally, in vivo test using an ototoxicity-induced animal model showed that Dopa-NPs improved hearing protection, as indicated by the auditory brainstem response (ABR) test. In conclusion, mucoadhesive Dopa-NPs exhibit enhanced drug delivery efficiency and safety, offering a promising strategy for inner ear drug delivery and chemotherapy.

**Graphical abstract:**

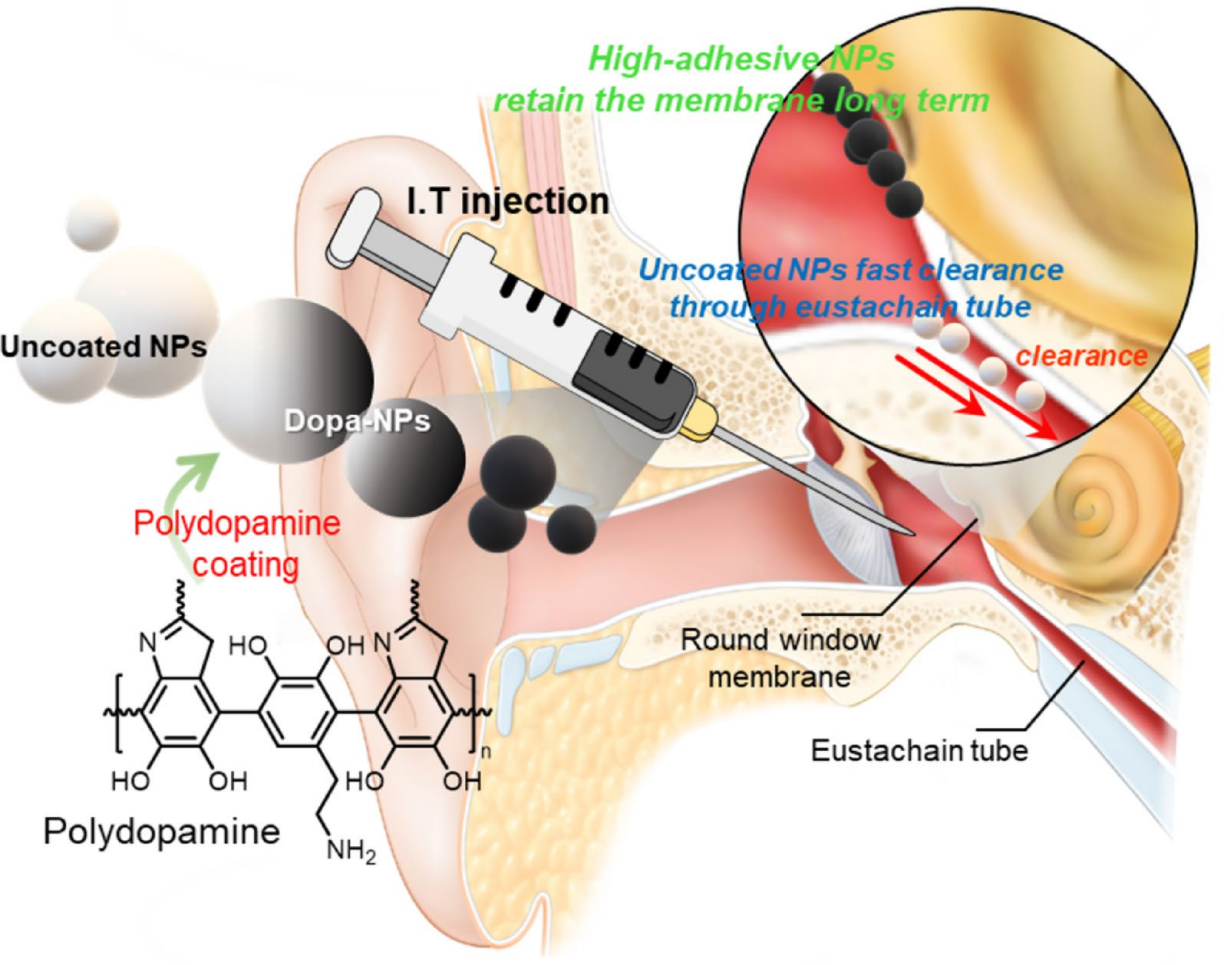

**Supplementary Information:**

The online version contains supplementary material available at 10.1186/s12967-025-07103-z.

## Introduction

Acute hearing loss poses a significant clinical challenge, highlighting the need for effective drug delivery strategies targeting the inner ear. Among the available pathways for drug transport from the middle ear to the cochlea, the round window membrane (RWM) is the principal entry route [1]. Intratympanic (IT) injection is the most widely used clinical approach to deliver drugs directly into the middle ear, increasing drug accumulation compared to oral or intravenous injection. However, the effectiveness of IT injections of a drug formula largely depends on prolonged drug retention in the middle ear and enhanced RWM permeability. The study by Salt et al. highlights the role of drug lipophilicity in round window membrane permeability, demonstrating that hydrophobic drugs exhibit enhanced penetration compared to hydrophilic counterparts [2].

Among the pharmacological agents used for its treatment, dexamethasone (Dex) stands out for its anti-inflammatory and immunosuppressive properties. However, the limited solubility of Dex poses hurdles in its delivery to the inner ear, prompting the exploration of novel delivery methods [3, 4]. Numerous strategies have been explored to formulate hydrophobic drugs for IT injection, and nanoparticle (NP)-based formulations have shown promising results [5, 6]. A study by Zhang et al. elucidated the translocation mechanism of polylactic-co-glycolic acid (PLGA)-NPs across the round window membrane, demonstrating their ability to penetrate *via* the transcellular pathway [7, 8]. Our group also developed PLGA-NPs and single emulsion NPs and reported significantly improved therapeutic results in animal models [9, 10].

However, drug delivery to the inner ear through IT injection still faces significant challenges owing to the rapid clearance of drugs through the eustachian tube, which reduces drug retention and efficacy in the inner ear. Enhancing the retention of drugs within the middle ear space is therefore a critical factor for successful inner ear drug delivery. Conventional formulations, including aqueous solutions, often suffer from insufficient contact with the RWM and are prone to drainage, leading to a loss of therapeutic efficacy [11, 12]. Previous studies have demonstrated that embedding drugs in delivery systems such as hydrogels or NPs can mitigate this issue by prolonging contact with the RWM and enabling sustained drug release [13, 14].

In the proteinaceous cuticles enveloping mussel byssal threads, strong binding based on the catechol groups of 3,4-dihydroxyphenylalanine (Dopa) has been discovered. These bindings serve as crucial cross-linking mediators, contributing to the remarkable mechanical properties exhibited by the material [15–17]. Furthermore, the binding is reversible and exhibits a strength comparable to that of covalent bonds [18]. Leveraging these properties, numerous researchers have engineered mussel-inspired biomaterials containing Dopa characterized by high mechanical performance and self-healing capabilities, thus demonstrating immense potential for various biomedical applications [19, 20].

In this context, we designed NPs having mucoadhesive properties that can carry hydrophobic drugs like Dex and can increase adhering ability to the middle ear mucosa, thus increasing inner ear drug delivery through the round window membrane [21]. We developed mucoadhesive NPs consisting of polyvinyl alcohol (PVA) and PLGA, and coated their surface with polydopamine containing Dopa to achieve enhanced inner ear drug delivery *via* IT injection. Compared to non-adhesive uncoated control NPs, differences in drug concentrations absorbed in the cochlea and the safety and therapeutic efficacy of the NPs were evaluated in an animal model, with the aim of contributing to the advancement of therapeutic strategies for acute hearing loss.

##  Materials and methods

### Materials

Poly vinyl alcohol (PVA, MW 31,000–50,000), dexamethasone (DEX), pararosaniline, periodic acid, acetic acid, mucin from porcine stomach, Dopamine, and phosphoric acid (HPLC grade) were purchased from Sigma-Aldrich (St. Louis, MO, USA). Poly (lactic-co-glycolic acid) (PLGA, 50:50, IV 0.4 dl/g, ester-terminated) was purchased from Polysciences (Warrington, USA). 1 M tris buffer was purchased from Biosesang (Youngin, Korea). Dichloromethane (DCM), acetonitrile (ACN; HPLC grade), and methanol (HPLC grade) were purchased from Duksan (Ansan, Korea). Coumarin 6 was purchased from Alfa Aesar, Inc. (Ward Hill, MA, USA).

### Preparation and characterization of nanoparticles (NPs)

NPs were prepared by a traditional method involving emulsion, diffusion, and evaporation, as described in previous studies [22, 23]. Briefly, PLGA (50 mg) and DEX (4 mg) were dissolved in 1 mL of DCM. The organic solution was added dropwise to 5 mL of 1% PVA solution (aqueous phase), and the o/w emulsion was homogenized by sonicating for 2 min in ice. Then, the solvent was evaporated by magnetic stirring at 50 °C, and NPs were obtained by centrifugation at 45,000 rpm for 15 min. The obtained NPs were lyophilized for storage. To prepare polydopamine-coated Dopa-NPs, Dopamine in D.W. (5 mg/mL) was added to the uncoated NP solution in 10 mM Tris buffer (pH 8.5). After stirring for 6 h, centrifugation and lyophilization were performed under the same conditions. For in vitro and in vivo imaging, NPs containing coumarin 6 were prepared. Coumarin 6 (1 mg) was used instead of DEX (4 mg).

The size and stability of the nanoparticles were determined using a Zetasizer Nano ZS90 (Malvern Instruments, UK). The stability test of NPs was carried out at 37 °C in phosphate buffer solution (PBS), pH 7.4, for 14 days. The morphological characteristics of the nanoparticles were determined by transmission electron microscopy (TEM, Hitachi, Tokyo, Japan).

The encapsulation efficiency and drug release of the NPs were investigated using HPLC (Waters Corporation, USA). A C18 reverse phase column, XBridge Premier BEH (130 Å, 2.5 μm, 4.6 × 150 mm, Waters), was used in HPLC for chromatographic separations. The mobile phase consisted of an isocratic mixture of phosphoric acid adjusted to pH 2.2 and acetonitrile (60:40, v/v). The NPs were incubated at 37 °C in PBS for 9 days and lysed in methanol. Encapsulation efficiency (EE) was calculated as EE(%) = (weight of drug loaded in NP/weight of drug feed for loading) × 100%.

The mucoadhesive property was determined as the amount of mucin-adsorbed Dopa nanoparticles in a certain time period. NP suspensions (2 mg/mL) were mixed with type III mucin solution (2 mg/mL), vortexed, and incubated at 37 °C for 1 and 24 h. After incubation, the suspensions were centrifuged at 5,000 g for 10 min, and free mucin was measured in the supernatant by a colorimetric method using Periodic Acid-Schiff staining [24].

In addition, for the direct measurement of mucin adhesion test, a 0.1% mucin solution was applied to the surface of a 12-well plate and incubated overnight to prepare mucin-coated surfaces [25]. The prepared mucin surfaces were then treated with coumarin-loaded nanoparticles, either before or after dopamine coating, and incubated for 30 min before removal. After washing with PBS for 30 min in a shaker, the samples were observed using a fluorescence microscope (Nikon, Japan).

###  In vitro and in vivo safety evaluation of NPs

The in vitro toxicity assessment utilized HEI-OC1 cells, which were cultured in Dulbecco’s modified Eagle’s medium supplemented with 10% fetal bovine serum and 50 U/mL recombinant mouse interferon-γ and maintained in a humidified 10% CO2 environment at 33 °C. The maximum safe dosage of NPs and Dopa-NPs was determined via the MTT assay following the manufacturer’s protocol (EZ-Cytox; Daeil Lab, Seoul, ROK).

After confirming the maximum safe dosage of NPs and Dopa-NPs during in vitro toxicity experiments, we conducted in vivo toxicity tests by administering the drugs into the middle ear of mice at the maximum safe concentration for NPs and Dopa-NPs and monitored changes in hearing ability for two weeks. The method of drug administration is detailed below.

### Middle ear administration of drugs via bulla approach

All animal experiments conducted on mice adhered to the National Research Council’s Guide for the Care and Use of Laboratory Animals after approval from the Institutional Animal Care and Use Committee of the Clinical Research Institute of Daejeon St. Mary’s Hospital, College of Medicine, The Catholic University of Korea (approval number: CMCDJ-AP-202 -00).

To ensure consistency in drug administration, IT injections were performed following a previously established protocol [10]. Male C57BL/6 mice (eight weeks old, 20–23 g) were sourced from Orient Bio (Seoul, Republic of Korea) and anesthetized via intraperitoneal injection of 30 mg/kg tiletamine/zolazepam (ZoletilV R, Virbac, Carros, France) and 10 mg/kg xylazine (RompunV R, Bayer, Leverkusen, Germany). Once anesthetized, the mice were positioned supine on a temperature-controlled heating pad. A midline incision was carefully made to expose the left bulla. Using fine forceps, a small perforation was created in the bulla, allowing for the injection of approximately 5 µL of either NPs solution or DOPA-NP solution using a 31-gauge insulin syringe. Following drug administration, the perforation was sealed by covering it with adjacent muscle tissue. To manage post-procedural pain, Rimadyl (1.0 mg/kg; Pfizer, Walton Oaks, UK) was administered, and Baytril (10 mg/kg; Orion, Hamburg, Germany) was given intraperitoneally once daily as a preventive measure against middle ear infections.

###  In vivo drug delivery efficacy evaluation of NPs

To assess the impact of mucoadhesiveness on inner ear drug delivery via IT injection, the lipophilic fluorescent dye coumarin was encapsulated into both uncoated nanoparticles (NPs) and Dopa-NPs. The prepared solutions were then administered into the middle ear of mice following the previously described method. Subsequently, at predetermined time points, the cochlea was harvested and fluorescence within the cochlea was compared to evaluate drug delivery efficacy. Cochleae were collected 1, 3, and 6 h after administration, crushed using Tissue Lyser II (Qiagen, Hilden, Germany) with 200 µL of absolute methanol to create lysates to analyze the fluorescence absorbed by the entire cochlear tissue. The lysates were incubated overnight at 4 °C to maximize fluorescent compound extraction. After incubation, samples were centrifuged at 13,200 rpm for 15 min at 4 °C, and 150 µL of the supernatant was carefully collected. The supernatants were then diluted with an equal volume of distilled water, and fluorescence intensity was quantified using a microplate reader (TECAN Infinite M Plex, Tecan Trading AG, Switzerland).

Subsequently, we directly observed the fluorescence absorbed by the cochlear tissue under a fluorescence microscope to compare the drug delivery efficacy of the NPs and Dopa-NPs. Using a low-magnification fluorescence stereo microscope (Nikon SMZ800N, Tokyo, Japan), we observed the overall absorption pattern of fluorescence within the cochlear tissue, whereas a confocal microscope (LSM5 Live Configuration Variotwo VRGB; Zeiss, Jena, Germany) was used at high magnification to specifically observe fluorescence absorption in the organ of the Corti region. The cochlear tissue was prepared as follows.

### The cochlear tissue preparation observed by fluorescence

We used cochleae collected one hour after drug injection into the middle ear of C57BL/6 mice. The cochleae were fixed in 4% paraformaldehyde (Merck, Darmstadt, Germany) for one hour, and decalcified in 5% ethylenediaminetetraacetic acid (EDTA, 0.3 M) for 12 h. The otic capsule of these cochleae was then removed using fine forceps to ensure minimal damage to the lateral wall as much as possible. Therefore, we obtained whole cochleae with the otic capsule removed, without separating each turn of the cochlea, for observation under a fluorescence stereomicroscope.

To enable visualization under a confocal microscope, whole-mount preparations of the organ of Corti were performed. Using a sharp-angled micro-scalpel, the bony and membranous labyrinths, along with the tectorial membrane, were carefully dissected to fully expose the organ of Corti. The excised samples were then placed on slides, stained with DAPI (Vector Laboratories, Burlingame, CA), and mounted using Vectashield mounting medium for imaging.

### In vivo dexamethasone delivery and treatment of hearing loss

Finally, we aimed to deliver dexamethasone to the inner ear using uncoated NPs and Dopa-NPs, compare their delivery efficiencies, and assess the resulting therapeutic effects. We encapsulated 1 mg of Dex into NPs and subsequently administered the drug into the middle ear using the method described above. Cochleae were collected at 1, 3, and 6 h post-surgery, following the same protocol as the evaluation of coumarin delivery. The level of dexamethasone in the cochlear lysates of mice was determined using a validated high-performance liquid chromatography-mass spectroscopy (LC-MS/MS) assay as described previously [26]. The dexamethasone concentration in each sample was determined using LC-MS/MS (1290 Infinity II/Qtrap 6500; Sciex, Washington, DC).

To evaluate the therapeutic effects of Dex-loaded NPs, an ototoxicity-induced animal model was utilized. Approximately 5 µL of DEX-loaded uncoated NPs or DOPA-NP solution was administered into the middle ear 4 h prior to ototoxic drug exposure. Ototoxicity was induced via intraperitoneal injection of kanamycin (1000 mg/kg; Sigma-Aldrich, St. Louis, MO), followed by furosemide (180 mg/kg; Sigma-Aldrich, St. Louis, MO), as described in Jung et al., 2022. Subsequently, auditory function was assessed using auditory brainstem response (ABR) testing to compare hearing outcomes across treatment groups.

###  Auditory brainstem response (ABR) testing

Animals were anesthetized with an intraperitoneal injection of 30 mg/kg tiletamine/zolazepam (ZoletilV R, Virbac, Carros, France) and 10 mg/kg xylazine (RompunV R, Bayer, Leverkusen, Germany). The active electrode was positioned at the vertex of the skull, while the reference electrode was placed beneath the left pinna, and the ground electrode was inserted under the contralateral ear. ABR thresholds were recorded in response to frequencies ranging from 8 to 32 kHz, along with click stimuli, focusing on the operated ear. TDT System-3 (Tucker Davies Technologies, Gainesville, FL, USA) hardware and software were used for ABR recording. The auditory stimuli consisted of computer-generated tone pips, including tone bursts at 8, 16, and 32 kHz, each lasting 4 ms with a rise-fall time of 1ms, in addition to clicks. Sound intensity was adjusted in 10-dB steps for both tone bursts and click stimuli near the threshold. ABR waveforms were analyzed using BioSig RP ver. 4.4.1, and statistical comparisons of ABR threshold differences among the experimental groups were conducted.

### Statistical analysis

All experiments were performed at least three times, and the data are expressed as mean ± standard deviation (SD). The Kruskal-Wallis test, followed by Dunn’s multiple comparison test, was employed to analyze the ABR threshold results. Statistical significance was set at *p* < 0.05.

## Results

###  Preparation and characterization of nanoparticles

**Scheme 1:**
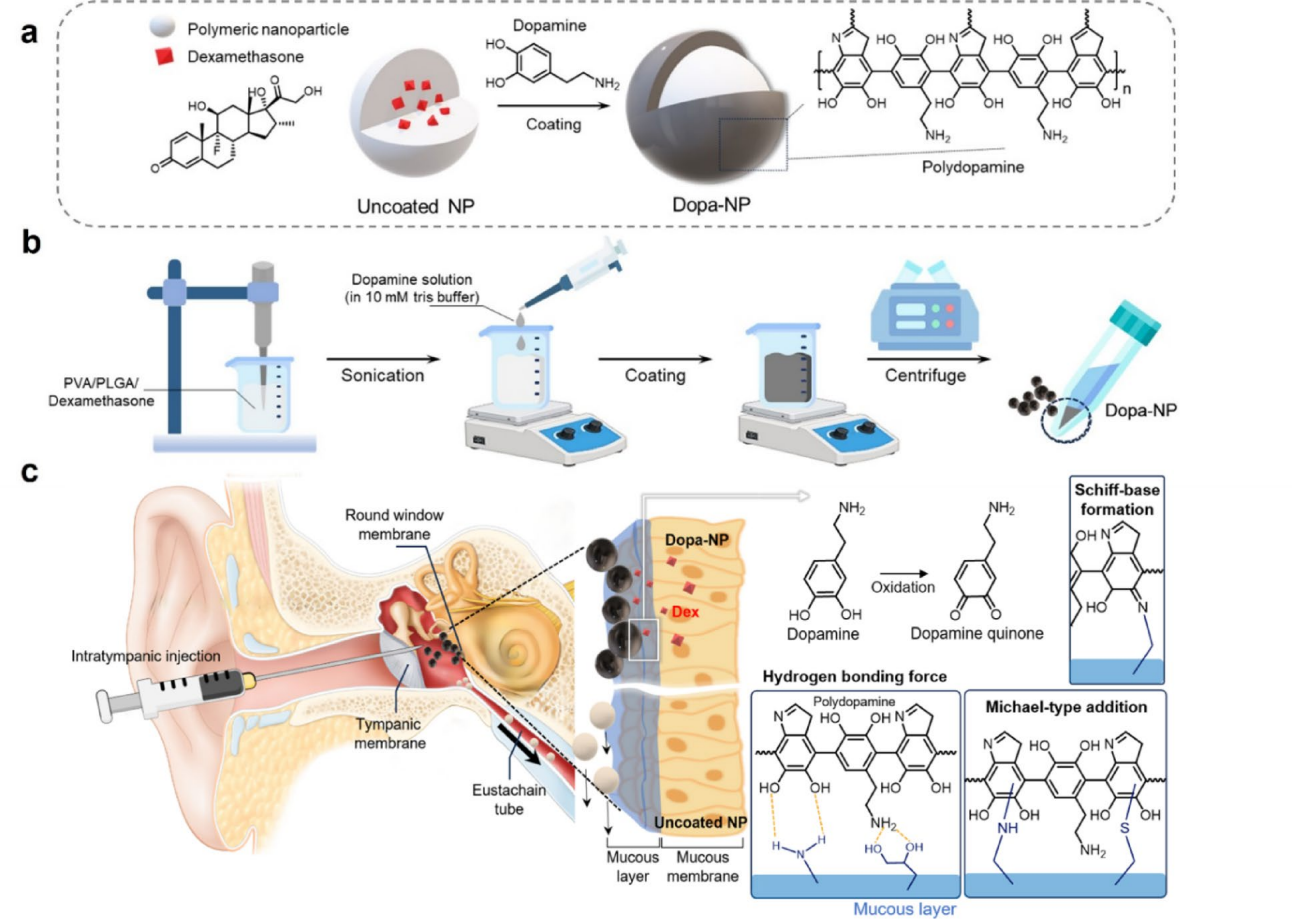
Schematic illustration of mucoadhesive polydopamine-coated nanoparticle-mediated inner ear drug delivery for hearing loss treatment. **A** Composition and chemical mechanism of DOPA-NPs. **B** The preparation process of DOPA-NPs. **C** An illustration depicting the movement of Dopa-NPs with enhanced mucoadhesive properties in the inner ear through the interaction between the polydopamine coating and mucin. Dopa-NPs in the inner ear. Dopa-NPs, with enhanced adhesive properties, remained on the round window membrane for an extended period, effectively delivering a large amount of the drug into the middle ear to enhance the therapeutic effect. In contrast, uncoated NPs are easily cleared from the ear through the eustachian tube

**Fig. 1:**
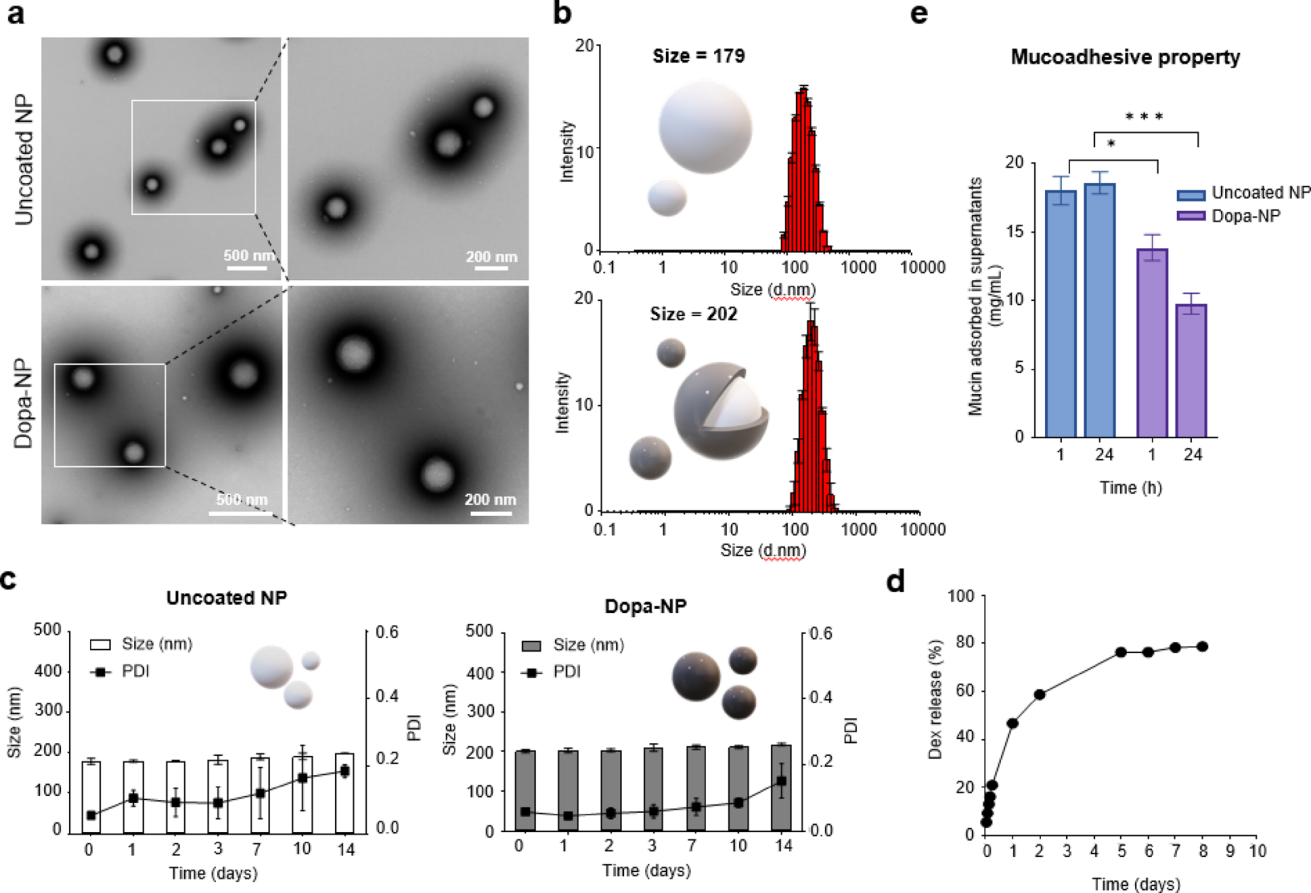
Characterization of uncoated NPs and dopa-NPs. **A** TEM images of uncoated NPs and DOPA-NPs. **B** Particle size distribution histogram of uncoated NPs and DOPA-NPs obtained using a zeta-sizer. **C** Stability data of NPs in PBS for 2 weeks. **D** In vitro drug release of uncoated NPs and DOPA-NPs was analyzed using HPLC. **E** Mucoadhesive properties assessed using Schiff’s reagent assay. This showed that DOPA-NPs would adhere to the mucosa more effectively than uncoated NPs

Considering the hydrophobicity of Dex, it was encapsulated in PLGA-NPs stabilized by PVA as a surfactant. Then, we coated the surface of the NPs with polydopamine to enhance their adhesiveness, resulting in Dopa-NPs. The coating process was achieved by wrapping Dopamine polymerized in a high-pH environment (Scheme 1). We expect that the NPs would adhere to the inner ear mucosa after IT injection, increasing their retention, preventing unintended drug loss, and improving drug delivery efficacy. The manufactured Dopa-NPs were visually characterized using TEM and SEM images, which showed that their spherical shape was well preserved after Dopa coating (Fig. 1a and Figure S1). The particle size, measured by a zeta-sizer, was 179 ± 2.9 nm before Dopa coating and increased slightly to 202 ± 1.6 nm after coating (Fig. 1b). Both NPs showed a narrow distribution and small polydispersity index (PDI) values, as shown in Figure S2. Furthermore, to assess the stability of the two particles, they were incubated in PBS, and it was observed that the particles remained stable for two weeks (Fig. 1c). The extent of dopamine coating on the surface of traditional PLGA-NPs was time-dependent, and the optimal conditions for subsequent experiments were determined. Dopa-NPs coated for 3 h did not exhibit significant color change, whereas a sufficient coating was observed under conditions of 5 h or longer (Figure S3). However, a longer coating process decreased the Dex content encapsulated in the particles (Table 1). After 3 h of dopamine coating, the amount of Dex encapsulated in NPs decreased by 29.20% compared to the pre-coating level. Further extension of the coating duration to 5 and 16 h resulted in a reduction of 47.70 and 73.60%, respectively. These findings suggest that excessive coating time can lead to unintended loss of the encapsulated drug, due to diffusion during the prolonged exposure to the coating solution. Accordingly, a 5-hour coating time was selected as the optimal condition to balance sufficient surface modification with minimal drug loss, and this condition was used for all subsequent experiments in this study. The encapsulation efficiency (EE) of Dex within the NPs was determined by HPLC (Figure S4 and S5). The EE was 45% in the uncoated NPs and decreased to 24% after the Dopa coating. The encapsulated drug exhibited a sustained release in vitro, with 13, 21, and 47% released after 3, 6, and 24 h, respectively (Fig. 1d and S6).

**Table 1:** Analysis of the amount of DEX in NPs during DOPA coating process by HPLC

Coating time (hour)	Dex value (before coating)	Dex value (after coating)	Reduction ratio (%)
3 h	1,407 ug/mL	996 ug/mL	-29.20%
5 h	1,369 ug/mL	716 ug/mL	-47.70%
16 h	1,289 ug/mL	341 ug/mL	-73.60%

Polydopamine in Dopa-NPs interacts with mucin, a key component of the ear mucosa. The catechol groups of dopamine are well known for enhancing chemical and physical adhesive properties. It primarily adheres to tissue surfaces by forming bonds with specific amino acid residues and thiol group of cysteine in the tissue mucosa via Michael addition reactions and Schiff base formation [18]. Moreover, strong binding stability is achieved through non-covalent interactions, such as hydrogen bonds, enabling robust fixation within the ear tissue and highly efficient drug delivery [27]. It is not based on the traditional biological ligands, it increased the binding of NPs similarly [28]. We performed a mucin-binding test to verify the adhesive properties of Dopa-NPs with mucin (Fig. 1e). Each NP sample was dispersed in a mucin solution, and NPs were removed from the solution after 1 and 24 h. The concentration of the remaining free mucin in the supernatant was analyzed using the Periodic Acid-Schiff staining method. As expected, lower amounts of free mucin were detected in the Dopa-NPs than in the uncoated control NPs, indicating that most mucin was bound to Dopa-NPs. This means that the Dopa coating significantly improved the adhesive ability of NPs. Consistently, the mucin-binding assay revealed a significantly higher fluorescence intensity for dopamine-coated nanoparticles compared to uncoated nanoparticles. The fluorescence quantification value of the uncoated nanoparticles was 603.122 ± 25.4 a.u., whereas the dopamine-coated nanoparticles exhibited a markedly increased value of 1269.96 ± 182 a.u., representing approximately a 2.1-fold enhancement (*p* < 0.01). These results demonstrate that the polydopamine coating significantly improves the mucoadhesive properties of the nanoparticles. (Figure S7).

### In vitro and in vivo safety evaluation of NPs

**Fig. 2:**
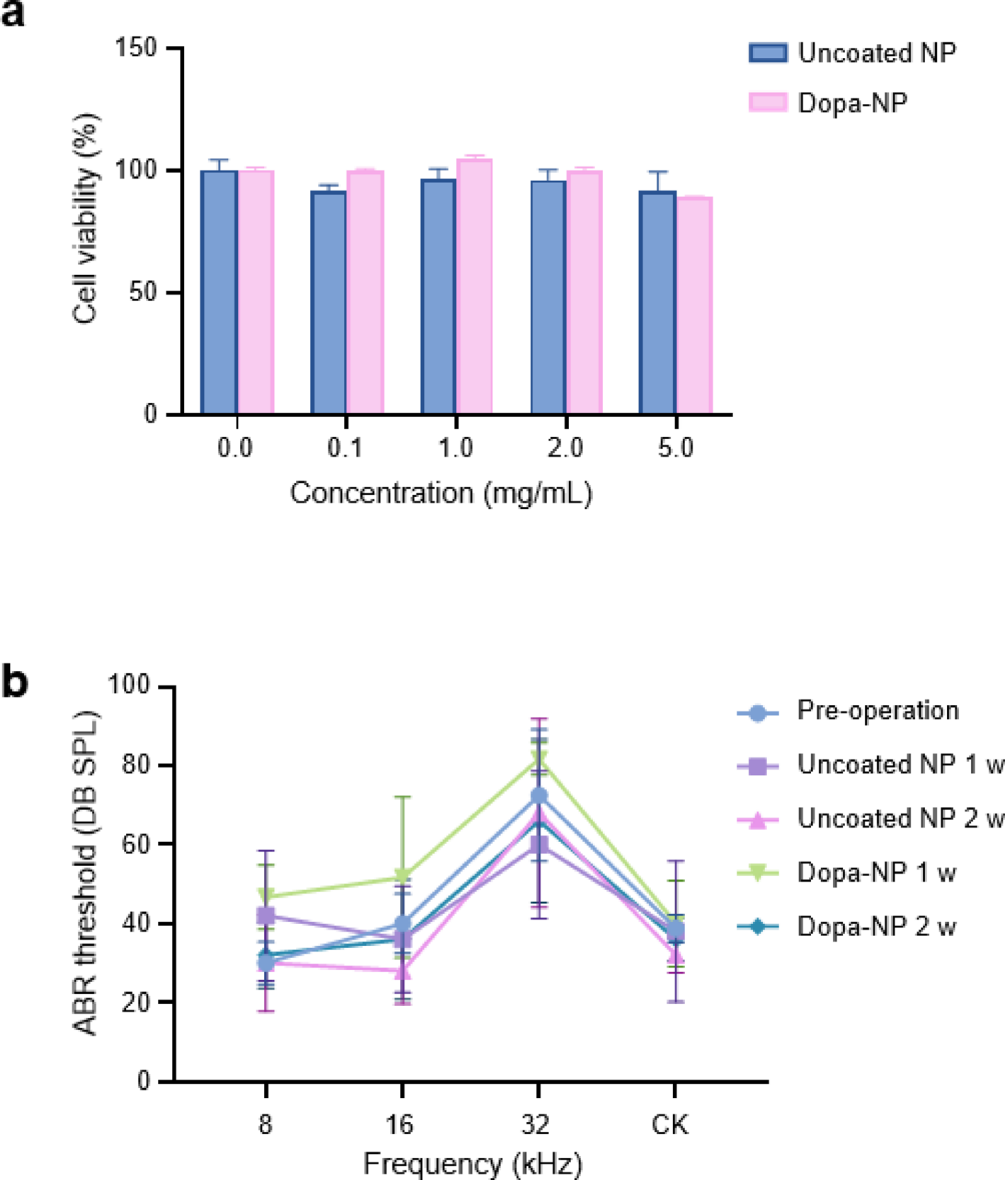
In vitro and in vivo safety evaluation of uncoated and DOPA-NPs. **A** Cell viability was assessed using the MTT assay and showed no cytotoxicity for both uncoated and DOPA-NPs. up to 5 mg/mL. **B** ABR test after IT injection (5 mg/mL) showed no significant hearing loss with uncoated and DOPA-NP-induced temporary threshold shift at 1 week, with recovery by 2 weeks

In *vitro* toxicity analysis was conducted using HEI-OC1 (House Ear Institute-Organ of Corti 1) cells to assess the cytotoxicity of uncoated and Dopa-NP. Both types of NPs exhibited no cytotoxicity at concentrations up to 5 mg/ml (Fig. 2a). This result indicates that both NPs do not introduce significant cytotoxicity at effective concentrations considering the therapeutic dosage, suggesting their suitability for further in vivo studies. For the in vivo safety evaluation, NPs were injected into the cochlea at a concentration of 5 mg/ml. Hearing was assessed using auditory brainstem response (ABR) measurements for 2 weeks post-injection. The results indicated no apparent hearing damage in both the NP and Dopa-NP groups when compared to the saline control group after two weeks (Fig. 2b). The Dopa-NP group exhibited a slight decrease in hearing ability one week post-injection, which was completely recovered by the two-week mark. This transient threshold shift observed at one week in the Dopa-NP group is likely attributable to the prolonged presence of mucoadhesive nanoparticles, which temporarily affected sound conduction. This suggests that both uncoated and Dopa-NPs are safe for use at the tested concentrations.

###  Inner ear distribution of NPs observed by in vivo fluorescence imaging

**Fig. 3:**
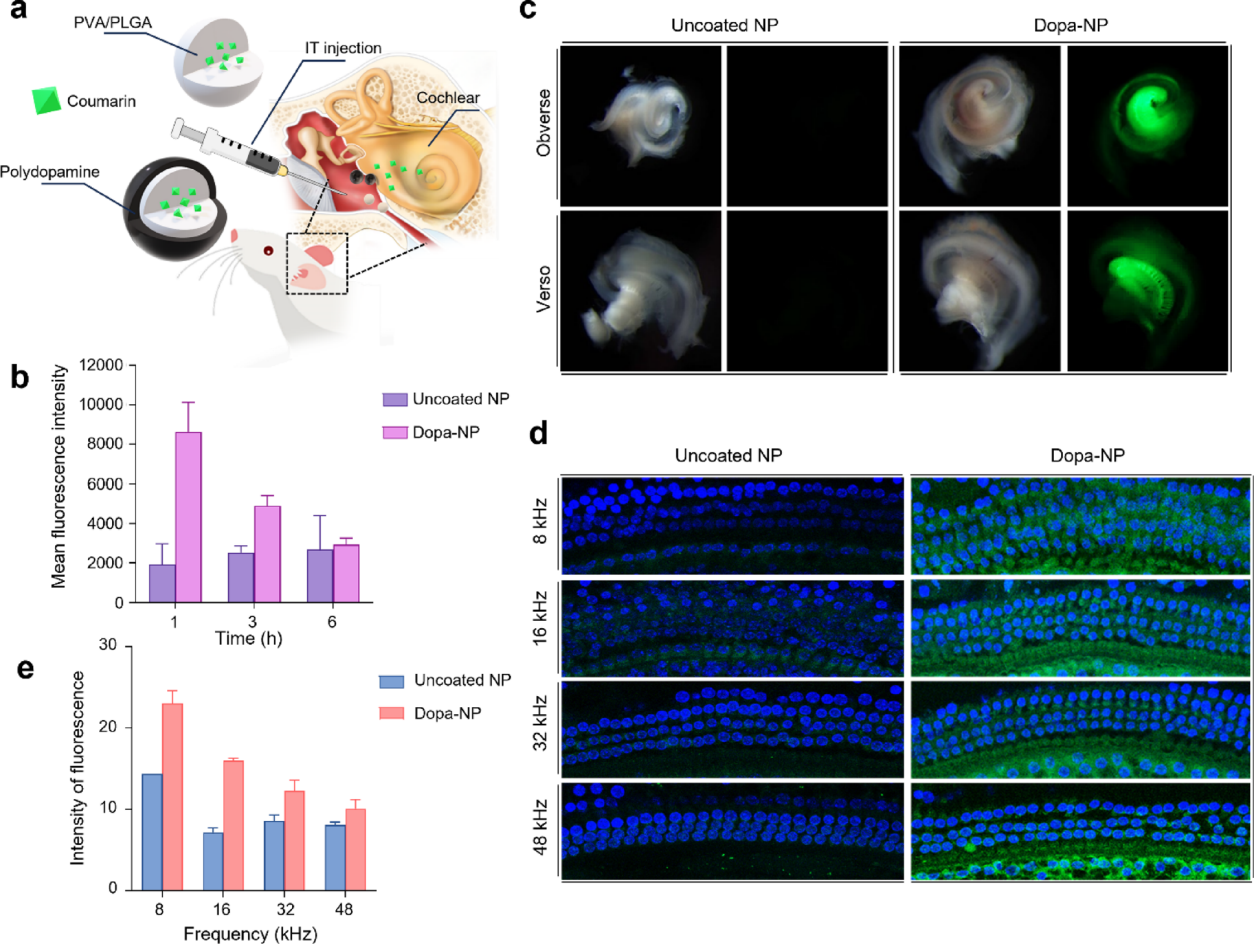
Fluorescence distribution and intensity of coumarin-loaded NPs in the cochlea. **A** Schematic representation of the IT injection of coumarin-loaded NPs into B6 mice. **B** Fluorescence intensity in cochlear homogenates at 1, 3, and 6 h post-administration showed higher uptake in the DOPA-NP group compared to uncoated NPs. **C** Fluorescence stereomicroscopy of cochleae harvested 1 h post-injection revealed stronger fluorescence in the modiolus with DOPA-NPs. **D** Confocal microscopy image and **E** Quantitative analysis of fluorescence intensity of the organ of Corti confirmed significantly higher fluorescence uptake in the DOPA-NP group across all frequencies

To evaluate the in vivo dispersion of NPs, we encapsulated lipophilic coumarin 6 dye within both NPs and then administered them to the middle ear cavity (Fig. 3a). Fluorescence intensity was measured in cochlear homogenates at 1, 3, and 6 h post-administration. The Dopa-NPs exhibited significantly higher fluorescence intensity than the uncoated NP group (*p* < 0.05), indicating enhanced drug dispersion and uptake facilitated by the mucoadhesive properties of Dopa (Fig. 3b).

Further analysis using fluorescence stereo-microscopy of cochleae harvested 1 h post-administration revealed intense fluorescence throughout the cochlea, with particularly high uptake in the modiolus, the central axis of the cochlea (Fig. 3c). In particular, to the best of our knowledge, this kind of imaging of the whole cochlea harvested has not been attempted in inner ear drug delivery studies until now. Additionally, confocal microscopy images of whole mounts of the organ of Corti demonstrated that the fluorescence intensity was markedly higher in the Dopa-NP group than in the uncoated NP group (Fig. 3d and e) in all areas. These results suggest that Dopa-NPs not only enhance overall cochlear distribution but also facilitate more efficient uptake in specific cochlear structures, such as the modiolus, organ of Corti, and apex, which is critical for effective inner ear drug delivery.

### Intracochlear Dex concentration analysis

**Fig. 4:**
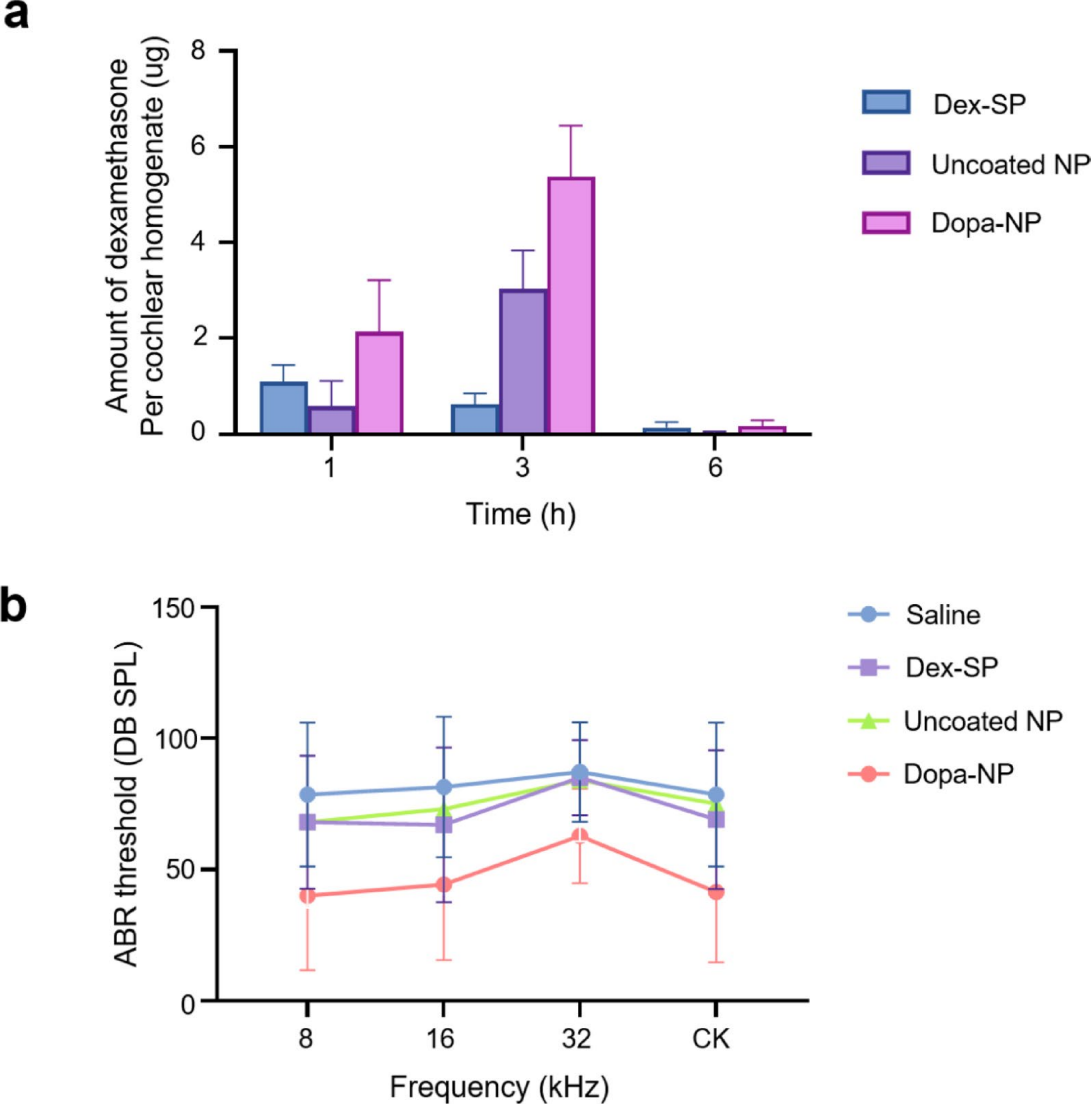
DEX concentration in cochlear homogenates and therapeutic efficacy in an ototoxic animal model. **A** LC-MS analysis of cochlear homogenates following IT administration of DEX (1 mg) as DEX-SP or DEX encapsulated in NPs. DOPA-NPs achieved higher cochlear drug concentrations than uncoated NPs or DEX-SP, with peak levels at 3 h post-injection. **B** ABR test in an ototoxic mouse model. Animals received uncoated NPs or DOPA-NPs containing 1 mg/mL dexamethasone 4 h before ototoxic drug administration. DOPA-NPs provided significantly better hearing protection at 1 week compared to the other groups

Then, Dex was encapsulated within NPs, and we assessed the intracochlear drug concentration of Dex after IT injection. Dopa-NPs demonstrated superior dexamethasone delivery compared with uncoated NP or the sodium phosphate form of Dex (Dex -SP), the currently used formulation of Dex in the clinic. These results underscore the efficacy of Dopa-NPs in effective and sustained drug delivery (Fig. 4a). The peak concentration of Dex was observed approximately 3 h post-injection in the Dopa-NP groups, which was different from the earlier peak (around 1 h) observed in coumarin fluorescence imaging (Fig. 3b). This is likely due to the difference in lipophilicity of Dex and coumarin dye, which influences their release, absorption, and distribution kinetics.

### Therapeutic efficacy evaluation in an ototoxic mouse model

To evaluate the therapeutic efficacy of Dopa-NPs, animals were treated with NP or Dopa-NP containing 1 mg/ml of Dex, followed by administration of ototoxic agents (1000 mg/kg of kanamycin and 180 mg/kg of furosemide) to induce hearing loss. Hearing ability was evaluated using the ABR test one week after the treatment. The results demonstrated significantly improved hearing outcomes in the Dopa-NP group compared with both the uncoated NP and control groups treated with Dex-SP (Fig. 4b). In particular, the Dopa-NP group showed superior hearing protection across all tested frequencies (8, 16, and 32 kHz), with a marked reduction in hearing loss, particularly at 32 kHz, which is known to be most sensitive to ototoxic damage. Overall, Dopa-NPs revealed the potential to deliver Dex more effectively, thus providing better initial protection against hearing loss than uncoated NPs and conventional Dex formulations.

##  Discussion

For effective inner ear drug delivery, a sufficient amount of the administrated drug should not be expelled through the eustachian tube. Therefore, we believe that ensuring strong adhesion to the RWM is a crucial factor. This study demonstrates the effectiveness of Dopa-coated NPs for inner ear drug delivery, particularly by improving their mucoadhesive properties. Dopa, which is derived from mussel adhesive proteins, has been widely recognized for its biocompatibility and low toxicity in various biomedical applications. The safety evaluation conducted in this study showed that Dopa-coated NPs exhibited no significant toxicity, both in vitro using HEI-OC1 cells and in vivo through IT injections in animal models. This aligns with previous studies on Dopa-functionalized materials, which have shown biocompatibility in various biological systems [29, 30]. During imaging with coumarin dye-loaded Dopa-NPs, intense fluorescence throughout the cochlea was observed, from the base to the apex, with particularly high uptake in the modiolus. They not only increased the overall cochlear distribution but also significantly enhanced the uptake of therapeutic agents in these key cochlear regions. This enhanced distribution and uptake are likely due to the strong mucoadhesive properties imparted by Dopa, which allows them to maintain prolonged contact with the cochlear mucosa, leading to sustained drug release [31, 32]. These properties are critical for achieving effective treatment outcomes, particularly in conditions such as in the cochlea.

Compared to conventional Dex-SP formulations, Dopa-NPs demonstrated significantly prolonged drug retention and improved therapeutic efficacy. The enhanced retention time and targeted delivery of Dopa-NPs suggest their potential to optimize treatment outcomes for inner ear disorders by reducing drug clearance and minimizing the need for repeated intratympanic administration. Our study also emphasizes the importance of considering the physicochemical properties of the drug and delivery vehicle in designing effective therapeutic strategies. These findings suggest that the lipophilicity of the drug, combined with the mucoadhesive properties of the delivery vehicle, can significantly influence the overall efficacy of the treatment. Future research should focus on optimizing the formulation of Dopa-NPs to enhance their drug loading capacity and release profiles, ensuring sustained and controlled delivery of therapeutic agents to the inner ear.

Another important aspect is that our strategy is about the surface coating of NP, not the NP itself, and is not limited to Dex-loaded PLGA-NPs. The facile Dopa coating for mucoadhesive properties and enhanced retention can be universally applied to various kinds of NPs for inner ear delivery. For example, NPs with other components, including metals, silica, exosomes, and carbon materials, can be easily coated with Dopa and injected into the inner ear [33]. Different kinds of drugs can also be delivered by Dopa-coated NPs. Furthermore, approaches with gene medicines, including mRNA and siRNA based on Dopa-NPs, would be interesting and promising in hearing loss treatment.

##  Conclusion

In summary, Dopa-coated nanoparticles can achieve sustained release through their enhanced mucoadhesive properties. Dopa-NPs exhibited in vivo and in vitro safety and enhanced drug delivery efficiency compared to PVA/PLGA nanoparticles. A single IT injection allows efficient drug delivery through the RWM, minimizing the risk of rapid clearance and maximizing therapeutic efficacy. The ease of administration of NPs by IT injection is also a significant advantage that is familiar to otolaryngologists. This study demonstrates the potential of mucoadhesive Dopa-NPs as an effective strategy for drug delivery to the inner ear by promoting adhesion to the middle ear mucosa, offering a promising avenue for advancing the treatment of inner ear disorders. Given the demonstrated safety and efficacy of Dopa-coated NPs, there is considerable potential for their application in the clinical treatment of sensorineural hearing loss and other inner ear disorders.

## Supplementary Information

Below is the link to the electronic supplementary material.

Supplementary Material 1

## Data Availability

The data that support the findings of this study are available from the corresponding author upon reasonable request.

## Abbreviations

NP: Nanoparticle, PVA: Poly vinyl alcohol
DEX: Dexamethasone
ABR: Auditory brainstem response
Dopa-NPs: Polymeric nanoparticles coated with polydopamine

## References

[1] Cervantes B, Arana L, Murillo-Cuesta S, Bruno M, Alkorta I, Varela-Nieto I. Solid lipid nanoparticles loaded with glucocorticoids protect auditory cells from Cisplatin-Induced ototoxicity. J Clin Med. 2019;8:1464. 31540035 10.3390/jcm8091464 PMC6780793

[2] Nyberg S, Abbott NJ, Shi X, Steyger PS, Dabdoub A. Delivery of therapeutics to the inner ear: the challenge of the blood-labyrinth barrier. Sci Transl Med 2019, 11. 10.1126/scitranslmed.aao0935 PMC6488020 30842313

[3] Chauhan AS. Dendrimers for drug delivery. Molecules. 2018;23:938. 29670005 10.3390/molecules23040938 PMC6017392

[4] Chen G, Hou SX, Hu P, Hu QH, Guo DD, Xiao Y. [In vitro dexamethasone release from nanoparticles and its pharmacokinetics in the inner ear after administration of the drug-loaded nanoparticles via the round window]. Nan Fang Yi Ke Da Xue Xue Bao. 2008;28:1022–4. 18583254

[5] Micaletti F, Escoffre JM, Kerneis S, Bouakaz A, Galvin JJ 3rd, Boullaud L, Bakhos D. Microbubble-assisted ultrasound for inner ear drug delivery. Adv Drug Deliv Rev. 2024;204:115145. 38042259 10.1016/j.addr.2023.115145

[6] Wang L, Zhang R, Jiang L, Gao S, Wu J, Jiao Y. Biomaterials as a new option for treating sensorineural hearing loss. Biomaterials Sci. 2024;12:4006–23. 10.1039/d4bm00518j 38979939

[7] Cardin V. Effects of aging and Adult-Onset hearing loss on cortical auditory regions. Front Neurosci. 2016;10:199. 27242405 10.3389/fnins.2016.00199 PMC4862970

[8] Choe WT, Chinosornvatana N, Chang KW. Prevention of cisplatin ototoxicity using transtympanic N-acetylcysteine and lactate. Otol Neurotol. 2004;25:910–5. 15547419 10.1097/00129492-200411000-00009

[9] Yang KJ, Son J, Jung SY, Yi G, Yoo J, Kim DK, Koo H. Optimized phospholipid-based nanoparticles for inner ear drug delivery and therapy. Biomaterials. 2018;171:133–43. 29689410 10.1016/j.biomaterials.2018.04.038

[10] Jung SY, Yoo J, Yang KJ, Jang SY, Yi G, Kim DK, Koo H. Intratympanic administration of alpha-lipoic acid-loaded pluronic F-127 nanoparticles ameliorates acute hearing loss. Nanomedicine. 2021;32:102329. 33181275 10.1016/j.nano.2020.102329

[11] Liu H, Hao J, Li KS. Current strategies for drug delivery to the inner ear. Acta Pharm Sinica B. 2013;3:86–96.

[12] Phuc Le T, Yu Y, Chan Kwon H, Shin S-A, Park Y-H, Moo Huh K. Novel self-degradable prodrug blend thermogel for intratympanic drug delivery to treat inner ear diseases. Chem Eng J. 2023;476:146726.

[13] Lajud SA, Nagda DA, Qiao P, Tanaka N, Civantos A, Gu R, Cheng Z, Tsourkas A, O’Malley BW Jr., Li D. A novel chitosan-hydrogel-based nanoparticle delivery system for local inner ear application. Otol Neurotol. 2015;36:341–7. 25587675 10.1097/MAO.0000000000000445 PMC4365436

[14] Piu F, Wang X, Fernandez R, Dellamary L, Harrop A, Ye Q, Sweet J, Tapp R, Dolan DF, Altschuler RA, et al. OTO-104: a sustained-release dexamethasone hydrogel for the treatment of otic disorders. Otol Neurotol. 2011;32:171–9. 21099726 10.1097/MAO.0b013e3182009d29

[15] Delaney DS, Liew LJ, Lye J, Atlas MD, Wong EYM. Overcoming barriers: a review on innovations in drug delivery to the middle and inner ear. Front Pharmacol. 2023;14:1207141. 37927600 10.3389/fphar.2023.1207141 PMC10620978

[16] Lin Q, Guo Q, Zhu M, Zhang J, Chen B, Wu T, Jiang W, Tang W. Application of nanomedicine in inner ear diseases. Front Bioeng Biotechnol. 2021;9:809443. 35223817 10.3389/fbioe.2021.809443 PMC8873591

[17] Zhang J, Tang H, Liu Z, Chen B. Effects of major parameters of nanoparticles on their physical and chemical properties and recent application of nanodrug delivery system in targeted chemotherapy. Int J Nanomed. 2017;12:8483–93. 10.2147/IJN.S148359 PMC5713688 29238188

[18] Bui HL, Su Y-H, Yang C-J, Huang C-J, Lai J-Y. Mucoadhesive, antioxidant, and lubricant catechol-functionalized Poly (phosphobetaine) as biomaterial nanotherapeutics for treating ocular dryness. J Nanobiotechnol. 2024;22:160. 10.1186/s12951-024-02448-x PMC11000383 38589911

[19] Mittal R, Pena SA, Zhu A, Eshraghi N, Fesharaki A, Horesh EJ, Mittal J, Eshraghi AA. Nanoparticle-based drug delivery in the inner ear: current challenges, limitations and opportunities. Artif Cells Nanomed Biotechnol. 2019;47:1312–20. 30987439 10.1080/21691401.2019.1573182

[20] Kaplan M, Öztürk K, Öztürk SC, Tavukçuoğlu E, Esendağlı G, Calis S. Effects of particle geometry for PLGA-Based nanoparticles: Preparation and in vitro/in vivo evaluation. Pharmaceutics 2023, 15. 10.3390/pharmaceutics15010175 PMC9862984 36678804

[21] Chandrasekhar SS, Rubinstein RY, Kwartler JA, Gatz M, Connelly PE, Huang E, Baredes S. Dexamethasone pharmacokinetics in the inner ear: comparison of route of administration and use of facilitating agents. Otolaryngol Head Neck Surg. 2000;122:521–8. 10740171 10.1067/mhn.2000.102578

[22] Staff RH, Landfester K, Crespy D. Recent Advances in the Emulsion Solvent Evaporation Technique for the Preparation of Nanoparticles and Nanocapsules. In *Hierarchical Macromolecular Structures: 60 Years after the Staudinger Nobel Prize II.* Edited by Percec V. Cham: Springer International Publishing; 2013: 329–344.

[23] Hoa LTM, Chi NT, Nguyen LH, Chien DM. Preparation and characterisation of nanoparticles containing ketoprofen and acrylic polymers prepared by emulsion solvent evaporation method. J Exp Nanosci. 2012;7:189–97.

[24] Zhang X, Wang Y, Zheng C, Li C. Phenylboronic acid-functionalized glycopolymeric nanoparticles for biomacromolecules delivery across nasal respiratory. Eur J Pharm Biopharm. 2012;82:76–84. 22659236 10.1016/j.ejpb.2012.05.013

[25] Crouzier T, Jang H, Ahn J, Stocker R, Ribbeck K. Cell patterning with mucin biopolymers. Biomacromolecules. 2013;14:3010–6. 23980712 10.1021/bm400447z PMC4076112

[26] Achberger K, Probst C, Haderspeck J, Bolz S, Rogal J, Chuchuy J, Nikolova M, Cora V, Antkowiak L, Haq W et al. Merging organoid and organ-on-a-chip technology to generate complex multi-layer tissue models in a human retina-on-a-chip platform. Elife 2019, 8. 10.7554/eLife.46188 PMC6777939 31451149

[27] Xu Y, Hu J, Hu J, Cheng Y, Chen X, Gu Z, Li Y. Bioinspired polydopamine hydrogels: strategies and applications. Prog Polym Sci. 2023;146:101740.

[28] Cavallaro PA, De Santo M, Belsito EL, Longobucco C, Curcio M, Morelli C, Pasqua L, Leggio A. Peptides targeting HER2-Positive breast cancer cells and applications in tumor imaging and delivery of chemotherapeutics. Nanomaterials (Basel) 2023, 13. 10.3390/nano13172476 PMC10490457 37686984

[29] Lee H, Scherer NF, Messersmith PB. Single-molecule mechanics of mussel adhesion. Proc Natl Acad Sci U S A. 2006;103:12999–3003. 16920796 10.1073/pnas.0605552103 PMC1559742

[30] Wei W, Yu J, Gebbie MA, Tan Y, Martinez Rodriguez NR, Israelachvili JN, Waite JH. Bridging adhesion of mussel-inspired peptides: role of charge, chain length, and surface type. Langmuir. 2015;31:1105–12. 25540823 10.1021/la504316q PMC4310636

[31] Chen Y, Gu J, Liu J, Tong L, Shi F, Wang X, Wang X, Yu D, Wu H. Dexamethasone-loaded injectable silk-polyethylene glycol hydrogel alleviates cisplatin-induced ototoxicity. Int J Nanomed. 2019;14:4211–27. 10.2147/IJN.S195336 PMC6559256 31239676

[32] Chowdary KP, Rao YS. Mucoadhesive microspheres for controlled drug delivery. Biol Pharm Bull. 2004;27:1717–24. 15516712 10.1248/bpb.27.1717

[33] Kim SB. Function and therapeutic development of exosomes for cancer therapy. Arch Pharm Res. 2022;45:295–308. 35604532 10.1007/s12272-022-01387-1 PMC9125016

