# Supplementary material for: Mucoadhesive polydopamine-coated nanoparticle-mediated inner ear drug delivery for hearing loss treatment
Source: J Transl Med. 2025 Oct 8;23:1066. doi: 10.1186/s12967-025-07103-z (PMC12505578; doi:10.1186/s12967-025-07103-z)
Supplement: Supplementary file 1 — Supplementary Material 1 [file 12967_2025_7103_MOESM1_ESM.docx]

Supporting Information

**Mucoadhesive polydopamine-coated nanoparticle-mediated inner ear drug delivery for hearing loss treatment**

Subin Kim ^a,1^, Seo Young Cheon ^b,1^, Keum-Jin Yang ^a^, Seong Su Won ^a^, Dong-Kee Kim ^a*^, Heebeom Koo ^b*^

^a^Department of Otolaryngology, College of Medicine, The Catholic University of Korea. Daeheung-dong, Jung-gu, Daejeon, Republic of Korea.

^b^Department of Medical Life Sciences and **Department of Medical Sciences** (Graduate School), College of Medicine, The Catholic University of Korea, 222 Banpo-daero, Seocho-gu, Seoul 06591, Republic of Korea


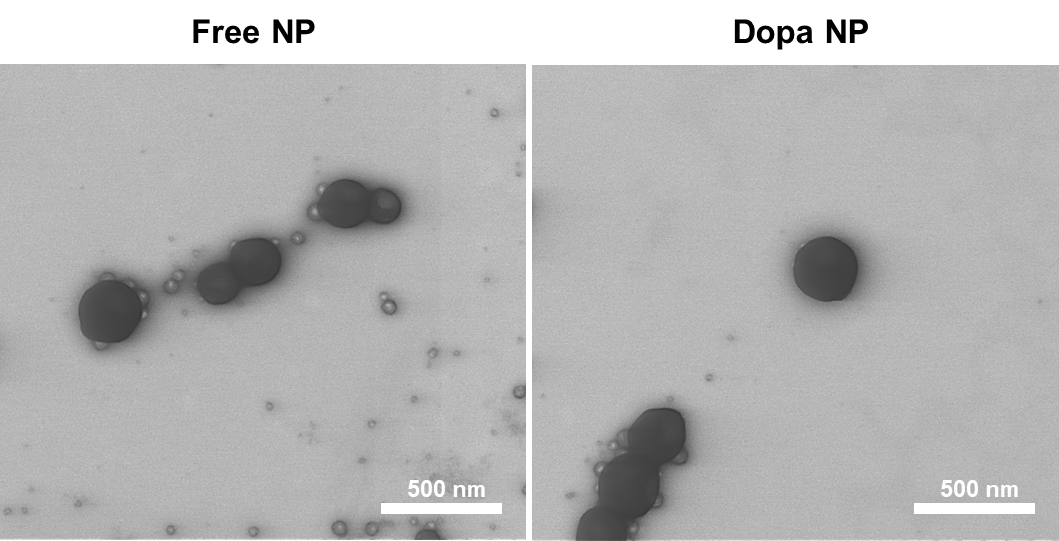


**Figure S1.** Scanning electron microscope (SEM) image of nanoparticles

**Figure S2.** PDI value of Free and dopa nanoparticle (NP).


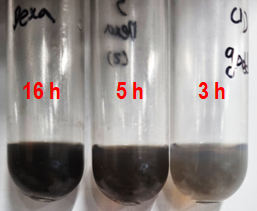


Figure S3. Image of Dopa NP over coating time

| 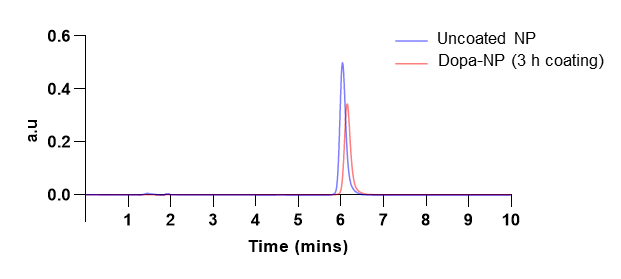 |
| --- |
| 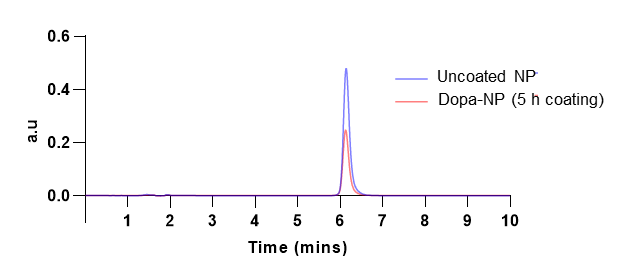 |
| 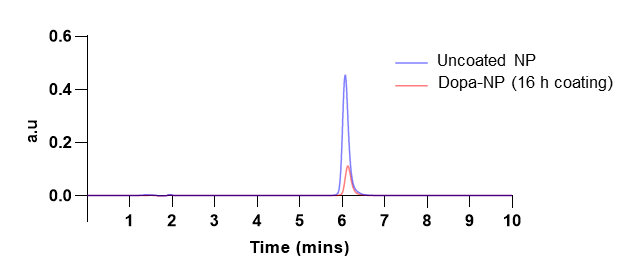  Figure S4. Analysis of dexamethasone content of uncoated NP and Dopa NP over coating time using HPLC |

**Figure S5.** Analysis of dexamethasone standard as concentrations (0.5, 0.25, 0.125, and 0.0625 ug/mL) by HPLC.

Figure S6. Analysis of encapsulation efficiency of nanoparticle by HPLC.

**
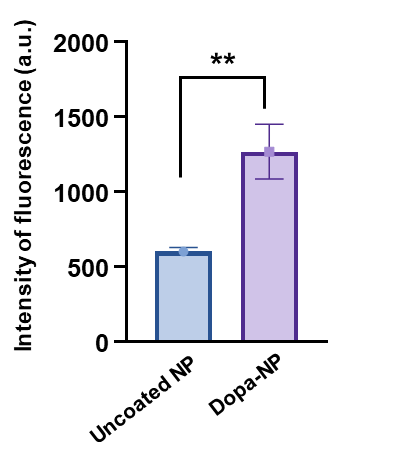
**

**Figure S7.** Adhesion test of uncoated NP and Dopa-NP into mucin surface
